# The biological function of m6A reader YTHDF2 and its role in human disease

**DOI:** 10.1186/s12935-021-01807-0

**Published:** 2021-02-16

**Authors:** Jin-yan Wang, Ai-qing Lu

**Affiliations:** Department of orthopeadics, Zhangjiagang Hospital of Traditional Chinese Medicine, Jiangsu 215600 Zhangjiagang, People’s Republic of China

**Keywords:** m6A, YTHDF2, Cancers, Non-cancers, Biological function, Up and downregulation, signaling pathways

## Abstract

N6-methyladenosine (m6A) modification is a dynamic and reversible post-transcriptional modification and the most prevalent internal RNA modification in eukaryotic cells. YT521-B homology domain family 2 (YTHDF2) is a member of m6A “readers” and its role in human diseases remains unclear. Accumulating evidence suggests that YTHDF2 is greatly implicated in many aspects of human cancers and non-cancers through various mechanisms. YTHDF2 takes a great part in multiple biological processes, such as migration, invasion, metastasis, proliferation, apoptosis, cell cycle, cell viability, cell adhesion, differentiation and inflammation, in both human cancers and non-cancers. Additionally, YTHDF2 influences various aspects of RNA metabolism, including mRNA decay and pre-ribosomal RNA (pre-rRNA) processing. Moreover, emerging researches indicate that YTHDF2 predicts the prognosis of different cancers. Herein, we focus on concluding YTHDF2-associated mechanisms and potential biological functions in kinds of cancers and non-cancers, and its prospects as a prognostic biomarker.

## Introduction

As far as we know, there are over 160 post-transcriptional modifications of RNA, which are characterized among all living organisms and produce a functional diversity that allows four basic ribonucleotide residues to obtain multiple functions [1]. Accumulating researches discovered a variety of RNA modifications in eukaryotic mRNAs, such as pseudouridine (Ψ), 5-methylcytosine (m5C), N1-methyladenosine (m1A) and m6A modifications [2]. m6A modification was first identified in mRNA-enriched RNA fractions in 1974 [3]. It was the methylation of the N6 position of adenosine bases and the most common internal RNA modification in eukaryotic cells [4]. With the application of available methods for detecting m6A, insights into the underlying mechanisms have been disclosed in recent decades. m6A RNA methylation is enriched in 3′untranslated regions (3′UTRs) [5, 6], and functions to modify kinds of RNAs, such as microRNAs (miRNAs) [7, 8], long non-coding RNAs (lncRNAs) [9] and messenger RNAs (mRNAs) [10].

m6A modification was recognized as a dynamic and reversible post-transcriptional modification in mammalian cells and maintained by multi-components, such as methyltransferase (MTase) complex and demethylase [11]. MTase complex referred to “writers”, including methyltransferase-like 3/14/16 (METTL3/14/16), KIAA1429, wilms tumor 1-associated protein (WTAP), RBM15 and RBM15B, which appended m6A sites and significantly influenced specific physiopathological processes [12]. Demethylase referred to as “erasers”, including alkB homolog 5 (ALKBH5) and fat mass and obesity-associated protein (FTO), acted to changeover the methylation [13, 14]. Besides, another group of m6A binding proteins, including YT521-B homology (YTH) domain family, IGF2BP1/2/3 and HNRNPA2B1, were termed as "readers" and functioned to recognize m6A sites [15–18].

Accumulating evidence showed that YTHDF2, as a member of m6A “readers”, played a significant role in multiple diseases, such as hematopathy and cancers [19–21]. Besides, significant progress had been made in fully understanding the role of m6A modifications in mRNA decay [22], pre- ribosomal RNA (rRNA) processing [23], and so on. These mechanisms were associated with diverse physiological and pathological processes, such as regulating inflammatory response [24], inducing pluripotent stem cells [25], adipogenesis [19]. Therefore, YTHDF2 might have a far-reaching influence on the development of human diseases, especially cancers.

**Fig. 1:**
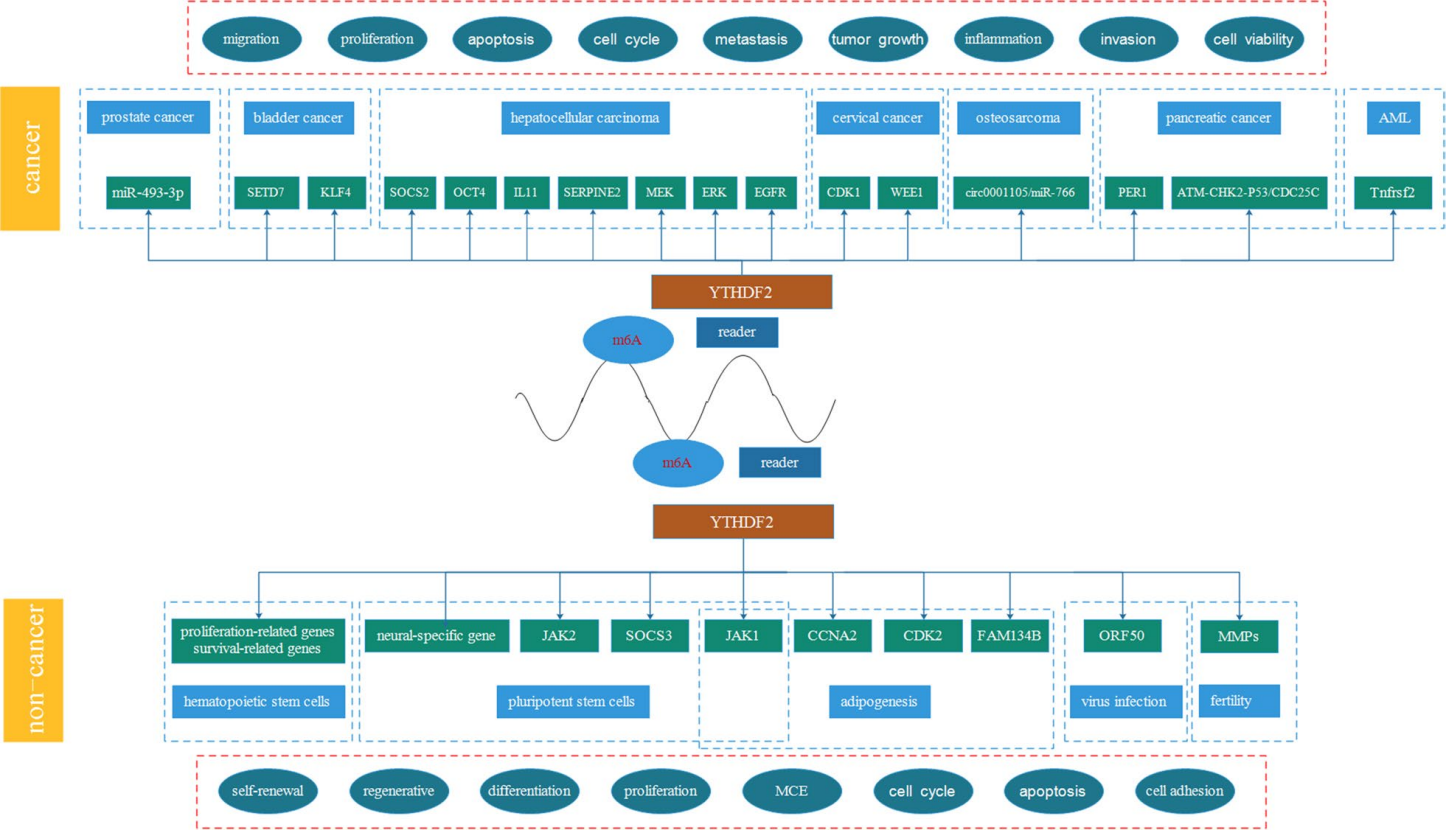
The role of YTHDF2 in human cancers and non-cancers. YTHDF2 took a great part in prostate cancer, bladder cancer, hepatocellular carcinoma, cervical cancer, osteosarcoma, AML and pancreatic cancer through modulating miR-403-3p, SETD7, KLF4, SOCS2, OCT4, IL11, SERPINE2, MEK, ERK, EGFR, CDK1, WEE1, circ0001105, PER1 and ATM-CHK2-P53/CDC25C. The modulation processes were closely related with tumor cell migration, invasion, metastasis, proliferation, apoptosis, cell cycle, cell viability and inflammation. In addition, YTHDF2 played an important role in hematopoietic stem cells, pluripotent stem cells, adipogenesis, virus infection, and male and female fertility by regulating proliferation-related genes, survival-related genes, neural-specific gene, JAK1/2, SOCS3, CCNA2, CDK2, FAM134B, Tnfrsf2, ORF50 and MMPs. The regulation process were strongly related with cell self-renewal, regenerative, differentiation, proliferation, MCE, cell cycle, apoptosis and cell adhesion

Herein, we concluded the biological functions and the role of YTHDF2 in diverse diseases, especially cancers (Fig. 1), and hoped to reveal the underlying mechanisms.

## The structure and mechanism of YTHDF2

**Fig. 2:**
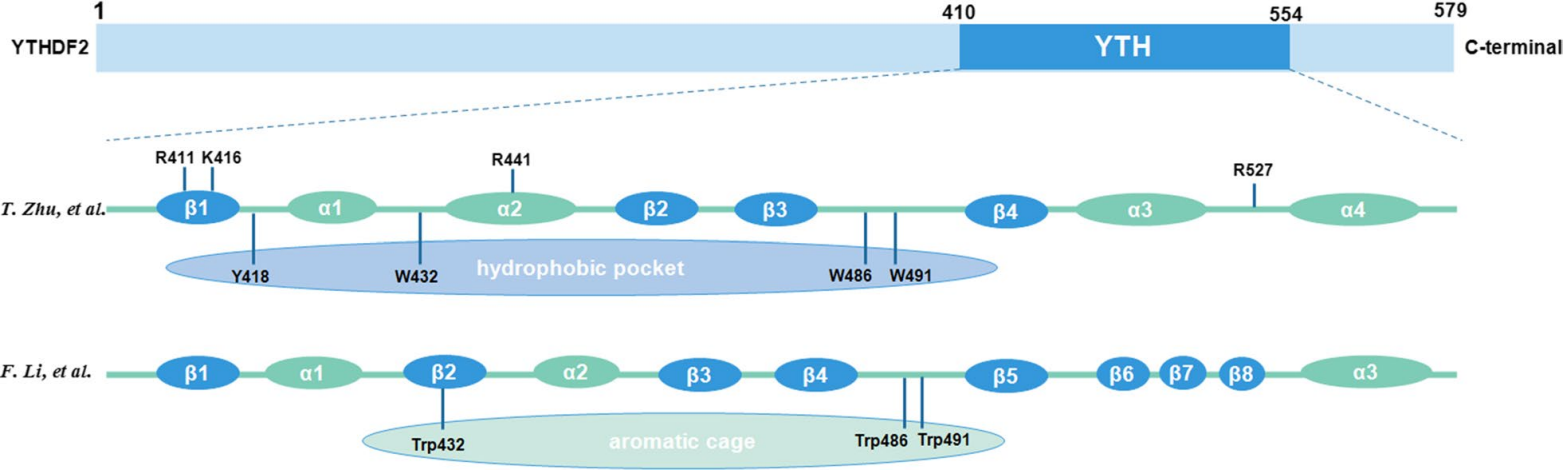
The structure of YTH domain in YTHDF2

The structure of YTHDF2 provided great help in disclosing the specific molecular mechanisms of the recognition of m6A sites. Zhu et al. [26] discovered that YTHDF2 consisted of a C-terminal YTH domain, which targeted the m6A-containing RNA. There were five known YTH domain containing proteins in human genome, including YTHDF1-3 and YTHDC1-2, and YTH domain is highly conserved in these YTH domain-containing proteins [27]. YTH domain of YTHDF2 was a globular fold with four-stranded β-sheets (β1-β4), four α helices (α1-α4) and flanking regions on two sides. Besides, residues K416 and R527 of the above C-terminal YTH domain greatly participated in targeting RNA backbone and residues W432 and W486 within the hydrophobic pocket. All these specific bindings contributed to the specific recognition of m6A. However, Li et al. [28] declaimed in the same year that YTH domain of YTHDF2 showed a globular fold with a central core of eight β strands (β1-β8), three α helices (α1-α3) and two 3_10_ helices. Additionally, the m6A mononucleotide was positioned in an aromatic cage, which consisted of residue Trp486 in β4-β5 loop, Trp432 in β2 strand and Trp491 in β4-β5 loop (Fig. 2).

Furthermore, YTHDF2 recruited the CCR4–NOT deadenylase complex. The modulation relied on the communication between YTHDF2 and the superfamily homology domain of the CNOT1 subunit, thus inducing the decay of m6A-containing mRNAs [29]. Besides, in vitro and in vivo assays suggested that m6A modification significantly enhanced the phase separation of YTHDF2 [30]. Liquid-like phase separation (LLPS) of YTHDF2 might depend on binding m6A mRNAs .

## The role of YTHDF2 in human cancers

In the field of cancer research, YTHDF2 had been found to greatly participate in the development of various cancers, including bladder cancer, hepatocellular carcinoma (HCC), gastric cancer, breast cancer, osteosarcoma, cervical cancer, prostate cancer, pancreatic cancer, acute myeloid leukemia (AML) and so on. It was interesting that YTHDF2 was up-regulated or down-regulated in different cancers, and played an oncogenic role or acted as a tumor suppresser (Table 1). Next, we will further discuss the specific role of YTHDF2 in multiple cancers.

**Table 1:** Expression, role, biological function and potential targets of YTHDF2 in cancers

Cancer	Expression	Role	Biological function	Target	Ref
Bladder Cancer	↑	Oncogene	Migration	SETD7 and KLF4	[21]
Hepatocellular Carcinoma	−	Oncogene		SOCS2	[33]
	−	Oncogene	CSC liver phenotype, metastasis	OCT4	[34]
	↓	Tumor suppressor	Inflammation, vasculature remodeling, metastasis	IL11, SERPINE2	[35]
	↓	Tumor suppressor	Proliferation, tumor growth	EGFR	[36]
Cervical Cancer	↑	Oncogene	Proliferation, apoptosis, cell cycle	-	[38]
Gastric Cancer	↑	Oncogene	Proliferation, cell cycle, apoptosis	-	[40]
Osteosarcoma	↓	Tumor suppressor	Cell viability, invasion, tumor growth		[42]
Pancreatic Cancer	−	Oncogene	Proliferation, migration, invasion Tumor growth, metastasis	PER1	[44]
Prostate Cancer	↑	Oncogene	Proliferation, migration	miR-493-3p	[46]

### YTHDF2 dysregulation in bladder cancer

Bladder cancer is the most common malignant tumor of the urinary tract and the treatment of bladder cancer has not been improved significantly since 30 years ago [31]. Recently, researchers constantly pay attention to the biological role of m6A modification in bladder cancer. Luckily, it was found that YTHDF2 was up-regulated in bladder cancer and the up-regulated YTHDF2 advanced the progression of bladder cancer through cooperating with METTL3 and directly degrading the mRNAs of SETD7 and KLF4 in an m6A dependent manner [21].

### YTHDF2 dysregulation in HCC

HCC, the main type of liver cancer, leads to the most common cancer-related death all over the world. Although surgical resection is the the best treatment for HCC, most patients are diagnosed at advanced stages with regional spread and metastasis, which are not eligible for surgical resection [32]. As a result, exploring the underlying mechanisms provides opportunities to improve the prognosis of HCC patients. Researches disclosed that YTHDF2 cooperated with METTL3 and took a part in HCC progression [33]. In detail, METTL3-mediated m6A modification repressed the suppressor of cytokine signaling 2 (SOCS2), which acted as a tumor suppressor in HCC via YTHDF2-mediated mRNA degradation. He et al. [34] discovered that YTHDF2 might function as an oncogene in the development of HCC. YTHDF2 advanced the cancer stem cell (CSC) liver phenotype and HCC lung metastases by adjusting the m6A methylation in the 5′UTR of OCT4 mRNA.

In contrast, some researches indicated that YTHDF2 severed as a tumor suppressor in HCC. For example, YTHDF2 was down-regulated in both hypoxic cells and HCC tissues, and inversely related to the m6A/A ratio [35]. Furthermore, hypoxia blocked the expression of YTHDF2 in a hypoxia-inducible factor-2α (HIF-2α)-dependent manner, and the deficiency of YTHDF2 promoted HCC cell growth, inflammation, metastasis and vasculature remodeling. Mechanistically, the above-mentioned functions were achieved by YTHDF2 through processing the decay of interleukin 11 (IL11) and serpin family E member 2 (SERPINE2) mRNAs. Zhong et al. [36] also declared that the inhibition of YTHDF2 was induced by hypoxia in HCC cells. And the expression of YTHDF2 was inversely correlated with HCC cell proliferation through activating MEK and ERK signaling pathways and destabilizing EGFR mRNA.

### YTHDF2 dysregulation in cervical cancer

Cervical cancer is one of the most prevalent gynecological cancers across the world. Surgical resection and chemo-radiotherapy is the main treatment for cervical cancer [37]. However, the specific mechanisms underlying the development of cervical cancer remain unclear. The expression of YTHDF2 was up-regulated in the cervical cancer tissues. In detail, YTHDF2 knockdown restrained proliferation, promoted apoptosis, and arrested the cells at the S phase in cervical cancer cells [38]. YTHDF2 silence in HeLa cells was correlated with a mitotic entry delay. This regulation partly depended on the activation of Wee1-like protein kinase (WEE1), a negative regulator of the cell cycle, and the stability of YTHDF2, which is greatly associated with cyclin-dependent kinase 1 (CDK1) activity [22].

### YTHDF2 dysregulation in gastric cancer

It is known that gastric cancer is one of the malignant cancers of the digestive tract [39]. In recent years, studies focus on the biological role of YTHDF2 in gastric cancer. YTHDF2 was found to be significantly up-regulated in gastric cancer tissues compared with that in normal tissues. In vitro assay revealed that YTHDF2 slience inhibited gastric cancer cell proliferation, arrested cell in G1 phase, and accelerated cell apoptosis [40].

### YTHDF2 dysregulation in osteosarcoma

Osteosarcoma is one of the most common malignant bone tumors in childhood and adolescence and the survival rate for patients with metastatic or relapsed osteosarcoma remains low at approximately 20% in over 25 years [41]. Recently, studies constantly pay attention to the role of YTHDF2 in the progression of osteosarcoma. YTHDF2 was found to cooperate with a circRNA in the development of osteosarcoma. Firstly, YTHDF2 was discovered to be down-regulated in osteosarcoma tissues [42]. Also, the findings offered evidence that YTHDF2 took a part in the tumorigenesis and progression of osteosarcoma through adjusting the circ_0001105/miR-766 axis.

### YTHDF2 dysregulation in pancreatic cancer

Pancreatic cancer is also a prevalent malignancy. On account of tumor metastasis and recurrence and lack of effective treatments, the clinical outcomes of pancreatic cancer remain poor [43]. One research found that YTHDF2 participated in the progression of pancreatic cancer via collaborating with ALKBH5, a pivotal demethylase of m6A [44]. The loss of ALKBH5 post-transcriptionally decreased the expression of PER1 in YTHDF2-dependent manner, and facilitated cell migration, invasion, proliferation and tumor growth in pancreatic cancer. Moreover, the up-regulation of PER1 contributed to reactivating the ATM-CHK2-P53/CDC25C signaling pathway, which greatly suppressed tumor cell growth.

### YTHDF2 dysregulation in prostate cancer

In America, prostate cancer is the second leading cause of death. The main treatment of prostate cancer contains surgical removal of the prostate, chemo-radiotherapy and hormonal therapy. However, the 5-years survival rate declines significantly for patients with metastatic prostate cancer [45]. In the study of YTHDF2, researchers found that YTHDF2 was frequently overexpressed in prostate cancer and YTHDF2 knockdown elevated m6A level and retained tumor cell proliferation and migration with the up-regulated miR-493-3p [46].

### YTHDF2 dysregulation in acute myeloid leukemia (AML)

AML is one of the most common and fatal forms of hematopoietic malignancies. With standard chemotherapies, only a small number of patients with AML survive more than 5 years [47]. In 2006, Nguyen et al. [48] first identified YTHDF2 in AML with reciprocal 21q22/RUNX1 translocations. AML was an aggressive clonal disorder, which blocked myeloid differentiation and self-renewing of leukemic stem cells (LSCs) [49]. Until 2019, Paris et al. [20] declared that YTHDF2 was overexpressed in human AML and the up-regulation of YTHDF2 played an important role in the development of LSC and the initiation and propagation of AML partly through shortening the half-life of multiple m6A transcripts which were closely related with LSC function. For instance, the tumor necrosis factor receptor Tnfrsf2 was closely related to the apoptosis of LSCs. On the other hand, the deficiency of YTHDF2 was proved to enhance hematopoietic stem cells (HSCs) activity and sensitize AML cells to tumor necrosis factor (TNF). As a result, YTHDF2 was supposed to be a novel and promising target in the treatment of hematological malignancy.

### YTHDF2 dysregulation with cancer prognosis

Based on current researches, it was found that the expression of YTHDF2 was closely associated with cancer prognosis. For example, up-regulated YTHDF2 indicated a poor prognosis in patients with cervical cancer [38]. And down-regulated YTHDF2 predicted more aggressive tumor phenotypes and a worse prognosis of osteosarcoma [42]. However, the relationship remains controversial even in the same cancer. He et al. [34] found that up-regulated YTHDF2 indicated poor overall survival and recurrence-free survival of HCC patients. However, Hou et al. [35] discovered that the down-regulation of YTHDF2 was significantly connected with poor TNM stage classification, overall survival and recurrence-free survival of HCC patients.

## The role of YTHDF2 in stem cells

### YTHDF2 dysregulation in HSCs

Hematopoietic stem cells were essential for life-long hematopoiesis and contributed to a subtle the balance between self-renewal and differentiation [50, 51]. The transplantation of human umbilical cord blood (hUCB) HSCs had been applied in hematologic, immune, and genetic diseases. However, HSCs in hUCB were scarce, which greatly limited their extensive application [52]. In 2018, Li et al. [53, 54] declared that YTHDF2 knockout in mice specifically increased the number of HSCs with no defects in their progenitor or lineage cells. In the further functional assay, it was found that the down-regulated YTHDF2 expanded functional hUCB HSCs without influencing lineage commitment, and YTHDF2 modulated hUCB HSCs self-renewal by m6A-dependent mRNA decay. In the same year, Wang et al. [55] revealed that YTHDF2 took a part in reading and processing m6A modification in HSCs. Furthermore, the deficiency of YTHDF2 decreased the degradation of mRNAs of proliferation/survival-related genes under hematopoietic stresses, thus boosting the regenerative capacity of HSCs.

### YTHDF2 dysregulation in pluripotent stem cells (PSCs)

Embryonic stem cells (ESCs) hold unprecedented promise for the treatment of disease, organ transplantation and so on. Induced PSCs (iPSCs) resembled ESCs in multiple biological processes [56]. However, on account of ethical issues, iPSCs failed to be extensively used. Later, researchers uncovered that porcine iPSCs (piPSCs) might be potential alternative stem cells, due to their great similarity in the human genome and physiological characteristics [57]. Recent studies disclosed YTHDF2 was overexpressed in iPSCs [58]. Further mechanism exploration found that YTHDF2 in iPSCs acted to destabilize a set of m6A-modified transcripts, which was closely connected with neural development and contributed to the loss of pluripotency and inhibited the expression of neural-specific genes.

Li et al. [59] demonstrated that YTHDF2 modulated neural stem/progenitor cell (NSPC) self-renewal, proliferation and differentiation capabilities and the generation of neurons via facilitating m6A-mediated degradation of neural development-specific mRNAs. In addition, METTL3 deficiency weakened self-renewal ability and induced differentiation of piPSCs through inhibiting YTHDF1-mediated JAK2 translation and blocking YTHDF2-dependent SOCS3 mRNA decay. These modulations eventually suppressed the activation of JAK2–STAT3 pathway and the transcription of KLF4 and SOX2 [25]. YTHDF2 was also declared to participate in the regulation of bone marrow stem cells (BMSCs) differentiating into adipocytes. METTL3 knockdown in porcine BMSCs (pBMSCs) inhibited adipogenesis and enhanced YTHDF2-dependent JAK1 mRNA stability [60]. JAK1 modulated adipogenesis via mediating signal transducer and activator of transcription (STAT)5 expression and activity, thus regulating the CCAAT/enhancer binding protein (C/EBP)βtranscription.

### YTHDF2 dysregulation in mesenchymal stem cells (adipogenesis)

M6A modification also greatly participated in adjusting adipogenesis, but the specific role of m6A on typical genes remained unclear [61]. Later, YTHDF2 was found to significantly participate in the recognition and degradation of methylated mRNAs, thus decreasing the expression of Cyclin A2 (CCNA2) and cyclin dependent kinase 2 (CDK2), which played essential roles in the mitotic clonal expansion (MCE) at the early stage of adipocyte differentiation [19]. CCNA2 and CDK2 were key cell cycle regulators, thereby promoting the cell cycle and suppressing adipogenesis of preadipocytes [62]. Moreover, YTHDF2 recognized and bound the m6A site of FAM134B to decrease the mRNA lifetime and inhibit the protein expression [63]. FAM134B was a cis-Golgi transmembrane protein and recognized to be greatly involved in preadipocytes adipogenic differentiation and lipid deposition.

## The role of YTHDF2 in human non-cancers

### YTHDF2 dysregulation in virus infection

Increasing studies indicated that m6A-related genes had pro- and anti-viral effects in distinct viral life cycles [64–66]. In 2018, Hesser et al. [67] discovered m6A level was up-regulated in cells infected with Kaposi’s sarcoma-associated herpes virus (KSHV). Mechanically, YTHDF2, which post-transcriptionally controlled the major viral lytic transactivator ORF50 abundance, mediated viral gene expression, lytic entry and virion production in KSHV-positive cell lines. However, the effect of m6A on KSHV infection relied on cell types.

### YTHDF2 dysregulation with human longevity

Cardelli et al. [68] identified a locus that was closely related to human longevity. It was up to the chromosomal regions with the highest density of Alu elements, in 1p35. Then they found the locus corresponded to a (TG)n microsatellite in the YTHDF2 gene, and suggested that this locus played a potential role in human longevity.

### YTHDF2 dysregulation with infertility

Male fertility relied on the mitosis of spermatogonia, which was a complex and highly controlled process [69]. YTHDF2 knockout in mouse spermatogonia acted to down-regulate matrix metallopeptidases (MMPs) and affected cell adhesion and proliferation through the m6A/mRNA degradation pathway [70]. As for female fertility, researchers claimed that YTHDF2, expressed at all stages of mammalian gametogenesis, was a pivotal factor in mammalian egg quality. In detail, the deficiency of YTHDF2 led to female infertility, as YTHDF2 was essentially needed for oocyte competence and regulated transcript dosage post-transcriptionally during oocyte maturation [71, 72].

## Discussion

In this review, we discussed the specific structure of YTHDF2 and the underlying mechanisms. In addition, YTHDF2 was dysregulated in human cancers, including bladder cancer, HCC, gastric cancer, breast cancer, osteosarcoma, cervical cancer, prostate cancer, pancreatic cancer, acute myeloid leukemia (AML) and so on. Although surgical treatment, chemoradiotherapy, immunotherapy and targeted therapy have significantly improve the prognosis of cancer patients, the underlying mechanisms of cancers remain unclear. This review summarized the current researches and concluded that YTHDF2 played an oncogenic role or acted as a tumor suppresser by regulating tumor cell proliferation, survival, growth, apoptosis, cell cycle, migration, invasion, metastasis, cell viability and so on,. These multiple effects were achieved through diverse pathways, such as collaborating with miRNAs or circRNAs, communicating with METTL3, and recognizing and decaying corresponding mRNA. However, the relevant reasons why YTHDF2 played opposite roles in different cancers were still unclear. Further researches might focus on the upstream molecular regulation mechanisms to explore the causes of YTHDF2 disorders in human cancers. Besides, regarding that YTHDF2 influenced various signaling pathways through recognizing m6A-modified sites of mRNAs, we’d better pay close attention to the most advantageous signaling pathways and get rid of those interfering ones. It was also declared that YTHDF2 was dysregulated in human non-cancers. For example, YTHDF2 took a great part in hematopoietic stem cell self-renewal and differentiation, inducing pluripotent stem cells, regulating adipogenesis, mediating viral gene expression, modulating male and female fertility. Further exploration might also focus on the detailed mechanisms regarding human non-cancers.

## Conclusion

In conclusion, YTHDF2 has great potential for clinical application by acting as a novel diagnostic/prognostic biomarker. However, further researches are still needed to expound the specific role of YTHDF2 in human cancers and non-cancers.

## Data Availability

Not applicable.

## Abbreviations

YTHDF2: YT521-B homology (YTH) domain family 2
rRNA: Pre-ribosomal RNA
m1A: N1-methyladenosine
m6A: N6-methyladenosine
m5C: 5-methylcytosine
3’UTRs: 3’untranslated regions
miRNAs: MicroRNAs
mRNAs: Messenger RNAs
lncRNAs: Long non-coding RNAs
MTase: Methyltransferase
METTL3/14/16: Methyltransferase-like 3/14/16
WTAP: Wilms tumor 1 (WT1)-associated protein
FTO: Fat mass and obesity-associated protein
ALKBH5: alkB homolog 5
m6A-RNA: m6A-containing RNA
SH: Superfamily homology
LLPS: Liquid-like phase separation
HCC: Hepatocellular carcinoma
SOCS2: Suppressor of cytokine signaling 2
OS: Overall survival
RFS: Recurrence-free survival
CSC: Cancer stem cell
HIF-2α: Hypoxia-inducible factor-2α
IL11: Interleukin 11
SERPINE2: Serpin family E member 2
WEE1: Wee1-like protein kinase
CDK1: Cyclin-dependent kinase 1
AML: Aacute myeloid leukemia
LSCs: Leukemic stem cells
TNF: Tumor necrosis factor
HSCs: Hematopoietic stem cells
hUCB: Human umbilical cord blood
ESCs: Embryonic stem cells
iPSCs: Induced pluripotent stem cells
piPSCs: Porcine induced pluripotent stem cells
NSPC: Neural stem/progenitor cell
BMSCs: Bone marrow stem cells
pBMSCs: Porcine BMSCs
STAT: Signal transducer and activator of transcription
C/EBP: CCAAT/enhancer binding protein
EGCG: Epigallocatechin gallate
CCNA2: Cyclin A2
CDK2: Cyclin dependent kinase 2
MCE: Mitotic clonal expansion
FAM134B: Sequence similarity 134 member B
KSHV: Kaposi’s sarcoma-associated herpes virus
MMPs: Matrix metallopeptidases
